# Wound Healing Activity of Nanoclay/Spring Water Hydrogels

**DOI:** 10.3390/pharmaceutics12050467

**Published:** 2020-05-21

**Authors:** Fátima García-Villén, Angela Faccendini, Dalila Miele, Marco Ruggeri, Rita Sánchez-Espejo, Ana Borrego-Sánchez, Pilar Cerezo, Silvia Rossi, César Viseras, Giuseppina Sandri

**Affiliations:** 1 Department of Pharmacy and Pharmaceutical Technology, Faculty of Pharmacy, University of Granada, Campus of Cartuja, 18071 s/n Granada, Spain; mcerezo@ugr.es (P.C.); cviseras@ugr.es (C.V.); 2 Department of Drug Sciences, Faculty of Pharmacy, University of Pavia, Taramelli Street 12, 27100 Pavia, Italy; angela.faccendini@gmail.com (A.F.); dalila.miele01@universitadipavia.it (D.M.); marco.ruggeri02@universitadipavia.it (M.R.); silvia.rossi@unipv.it (S.R.); g.sandri@unipv.it (G.S.); 3 Andalusian Institute of Earth Sciences, CSIC-UGR, Avenida de las Palmeras 4, Armilla, 18100 Granada, Spain; ritaespejo@hotmail.com (R.S.-E.); anaborrego@iact.ugr-csic.es (A.B.-S.)

**Keywords:** sepiolite, palygorskite, spring water, hydrogel, fibroblast, biocompatibility, wound healing

## Abstract

Background: hydrogels prepared with natural inorganic excipients and spring waters are commonly used in medical hydrology. Design of these clay-based formulations continues to be a field scarcely addressed. Safety and wound healing properties of different fibrous nanoclay/spring water hydrogels were addressed. Methods: in vitro biocompatibility, by means of MTT assay, and wound healing properties were studied. Confocal Laser Scanning Microscopy was used to study the morphology of fibroblasts during the wound healing process. Results: all the ingredients demonstrated to be biocompatible towards fibroblasts. Particularly, the formulation of nanoclays as hydrogels improved biocompatibility with respect to powder samples at the same concentration. Spring waters and hydrogels were even able to promote in vitro fibroblasts motility and, therefore, accelerate wound healing with respect to the control. Conclusion: fibrous nanoclay/spring water hydrogels proved to be skin-biocompatible and to possess a high potential as wound healing formulations. Moreover, these results open new prospects for these ingredients to be used in new therapeutic or cosmetic formulations.

## 1. Introduction

Chronic wounds are a current health problem with devastating consequences for patients and contribute to major costs to healthcare systems and societies. This type of wound results from an impaired wound healing process and is usually characterized by prolonged or excessive inflammation, persistent infections and inability of the dermal and/or epidermal cells to respond to repair stimuli [1,2,3]. The USA total Medicare spending for all wound types has been estimated to range from $28.1 to $96.8 billion. Diabetic foot ulcers (one of the main chronic wounds) accounted for $6.1 to $18.7 billion [2], the main cost burden attributed to amputations [1]. The development and implementation of new wound healing management strategies and healthcare products is, therefore, imperative. In recent years, different technological strategies have been proposed, including clays, metals, polymers and lipid-based systems among others, as reviewed by Bernal-Chávez et al. and García-Villén et al. [4,5]. Particularly, clay-based dressings have been proven to be useful in wound healing [5,6,7]. Among the different clay-based formulations, those composed of a clay suspended in mineral medicinal water, known as therapeutic muds, are widely used in clinical medical hydrology [8,9,10]. The solid phase of these systems is frequently incorporated into spring water to obtain a semisolid formulation known as “artificial thermal mud” [11,12,13,14]. Thermal muds have demonstrated their clinical effectiveness against dermatological affections such as psoriasis [15,16,17,18], atopic dermatitis, vitiligo [19,20], seborrheic dermatitis, fungal infections, eczema [18,21,22,23] and acne [24]. These clinical effects have been traditionally associated with the liquid phase. Avène and La Roche-Posay spring waters increase the fluidity of plasma membranes on cultured human skin fibroblasts [25,26,27,28] and have been useful in the management of chronic inflammatory skin diseases. La Roche-Posay spring water protected cultured human skin fibroblasts against lipid peroxidation induced by ultraviolet A and B radiation [29]. Boron and manganese-rich thermal waters are used for the treatment of ulcers and chronic wounds [30,31,32,33,34].

The influence of the thermal mud’s solid phase in the resulting clinical efficacy has not been studied in depth. The inorganic solid phase of thermal muds is mainly composed of clay minerals [8,10,35,36,37]. The clay mineral presence in wound healing formulations is supported by their already demonstrated biocompatibility with different types of skin cells [38,39,40]. The combination of clay minerals with other ingredients, such as polymers, allows the formation of scaffolding materials. In these occasions, clay minerals not only improved the mechanical strength and functionality of the polymers, but they also acted as synergistic ingredients for wound healing [41,42,43,44]. Biocompatibility of clay minerals such as halloysite, montmorillonite, palygorskite, sepiolite and imogolite have been widely studied [45,46,47,48,49,50,51,52,53,54,55]. Sasaki et al. reported that Mg^2+^ and Si^4+^ ions released by a synthetic Mg-rich smectite clay mineral can promote collagen formation and angiogenesis on skin wounds [56].

Moreover, palygorskite (“attapulgite”) has been used as scaffolding material when included in poly(lactic-co-glycolic acid) nanofibers, being crucial for mesenchymal cell adhesion and proliferation [57]. Sepiolite and palygorksite inhibit lipid peroxidation and possess anti-inflammatory properties by reducing neutrophil migration and edema [58,59]. Pharmaceutical grade palygorskite (Pharmasorb^®^ colloidal) did not only demonstrate to be biocompatible but also to protect fibroblasts from carvacrol cytotoxicity [6].

As discussed, both spring waters and clay minerals have been separately studied as potential wound healing ingredients. Synergistic effects would be expectable when both ingredients are formulated as spring-water/clay hydrogels. The role of these systems in wound healing studies has been scarcely addressed. The existing studies include a clinical study of diabetic gangrenous wounds treated with volcanic deposits muds [60], black-mud Dead Sea effects in wounded mice [61] and wound healing activity of emulsions prepared with a Brazilian clay [62].

With these premises, spring water hydrogels have been recently formulated and characterized, including mineralogical and chemical composition as well as textural and thermal properties, as a first step in the design of pharmaceutical-grade systems [63]. The second step would involve the study and evaluation of their biocompatibility. In this regard, the simplest starting point would include in vitro biocompatibility studies over skin cells like fibroblasts and in vitro wound healing studies.

Particularly, the in vitro biocompatibility and cell gap motility (wound healing) properties of two selected Spanish medicinal waters (obtained from Graena and Alicún de las Torres thermal stations), two clay minerals (palygorskite and sepiolite) and their corresponding hydrogels were studied. Additionally, particle size distribution and zeta potential measures as well as cation exchange capacities of the solid phases were carried out. In vitro wound healing tests were also evaluated by analyzing fibroblast F-actin microfilament organization by means of phalloidin staining. To the best of the authors’ knowledge, this is the first time that nanoclay/spring water mineral medicinal hydrogels have been evaluated in terms of in vitro cytotoxicity and wound healing.

## 2. Materials and Methods

### 2.1. Materials

Two pharmaceutical grade clays granted by TOLSA company (Madrid, Spain)—magnesium aluminum silicate (PS9) and attapulgite (G30)—with mineralogical identification previously evaluated (Table 1) were used [63].

Two medicinal waters from Graena (GR) and Alicún De Las Torres (ALI) thermal stations were used. Both spring sources are located in Granada (Andalusia, Spain) and are classified as hypothermal (ALI) and hyperthermal (GR), both of them having a strong mineralization [64,65]. Their pH, conductivity and elemental compositions have been determined in a previous study, and results are summarized in Table 2.

According to previous studies and characterizations [63], hydrogels were prepared mixing 10% *w*/*w* of each clay mineral with the corresponding spring water, by means of a turbine high-speed agitator (Silverson LT, Chesham, UK) at 8000 rpm for 10 min. The obtained hydrogels are summarized in Table 3.

### 2.2. Characterization of Inorganic Ingredients

#### 2.2.1. Particle Size

Particle size distribution was determined by a Malvern Mastersizer 2000 LF granulometer (Malvern InstrumentsTM). Measurements were performed in purified water after dispersing the solids by midst sonication for 30 s. The amount of sample added in each experiment was determined by the real-time laser obscuration degree indicated by “Mastersizer 2000” software. The optimal laser obscuration was delimited between 10–20%. Three replicates were performed for each sample, and statistical particle diameters (d_10_, d_50_, d_90_) were calculated together with the SPAN factor as an index of the amplitude of particle size distribution calculated according to Equation (1).

(1) $$\mathrm{SPAN}=\frac{d_{90}{-d}_{10}}{d_{50}}$$

#### 2.2.2. Cation Exchange Capacity

Cation exchange capacity (CEC) of PS9 and G30 was determined by dispersing 1 g of dry powder in 25 mL of tetramethyl ammonium bromide (TMAB) aqueous solution (1 M). The resultant dispersions were shaken in a Roller Mixer for 24 h and subsequently centrifuged (8000 rpm, 30 min) and filtered (0.45 µm pore size, HAWP-Millipore filter). Three replicates were performed for each sample together with two blanks of pure TMAB (1 M). The CEC determination is based on the exchange between bromine (in the TMAB solution) and cations present in the clay. The main exchangeable cations released from the clay mineral, once the Br exchanged, are K^+^, Na^+^, Mg^2+^ and Ca^2+^. Cations released, present in the resultant solution, were determined by ICP-OES (Optima 8300 ICP-OES Spectrometer, Perkin Elmer, Waltham, MA, USA), and CEC was calculated as the sum of exchangeable cations, expressed in mEq/100 g of clay mineral.

#### 2.2.3. Zeta Potential

Zeta-potential (ζ-potential) measurements determine the electrical potential difference between the stationary layer of fluid surrounding the solid particles and the bulk (electric double layer). Zeta potentials of different concentrations of PS9 and G30, suspended in Dulbecco’s modified Eagle medium, supplemented with 10% fetal bovine serum, 200 IU/mL penicillin and 0.2 mg/mL streptomycin, were also measured. Afterwards, ζ-potentials of the aforementioned suspensions were determined by using an electrophoretic light scattering (ELS) Zetasizer Naso-ZTM (Malvern Instruments, Worcestershire, UK). Samples were placed inside a folded capillary zeta potential cell (DTS1061, Malvern Instruments). Three replicates were obtained for each sample, and analyses were done at 25 ± 0.5 °C. During the analyses, 20 points were collected for each replicate and results expressed in mV.

### 2.3. In Vitro Tests of Inorganic Ingredients, Spring Waters and Hydrogels

#### 2.3.1. Biocompatibility Tests

Normal human dermal fibroblasts (NHDFs) from juvenile foreskin (PromoCell GmbH, Heidelberg, Germany) were used. All cells were between the 10th and 13th passages. NHDFs were grown in Dulbecco’s modified Eagle medium (DMEM, Sigma Aldrich^®^-Merck, Milan, Italy), supplemented with 10% fetal bovine serum (FBS, Euroclone, Milan, Italy), 200 IU/mL penicillin and 0.2 mg/mL streptomycin (PBI International, I), kept at 37 °C in a 5% CO_2_ atmosphere with 95% relative humidity (RH). Fibroblasts were seeded in 96-well plates (area 0.34 cm^2^/well) at a density of 10^5^ cells/cm^2^. Cells were grown 24 h to obtain sub-confluence. Then, cell substrates were washed with saline solution, and the cell substrates were put in contact with the samples. Biocompatibility of all samples was assessed after 24 h contact between samples and NHDF cultures. Powdered PS9 and G30 clay minerals were used in concentrations of 1000, 500, 50 and 5 μg/mL. ALI and GR medicinal waters were used in concentrations 0.25, 2.5, 25 and 50% *v*/*v*. Regarding PS9ALI, PS9GR, G30ALI and G30GR, cell contact concentrations were prepared in order to have equal amounts of clay mineral with respect to powdery samples, bearing in mind that hydrogels were prepared with 10% *w*/*w* of the corresponding clay. Since the final amount of clay fibroblasts was put in contact with the same as in the experiments with pristine clays, the same concentration codes (1000, 500, 50 and 5 μg/mL) were used. Briefly, clay and hydrogel samples were dispersed in sterile Hanks’ Balanced Salt Solution (Sigma-Aldrich) and mixed with Ultra-Turrax^®^ (S 25N, -18G, IKA, Staufen, Germany) for 5 min, 150,000 rpm. These initial suspensions were subsequently diluted in order to obtain samples having a concentration in the range previously mentioned. Eight replicates were assessed for all samples and for the control (NHDF cultures in pure DMEM phenol red).

After the 24 h contact, growth medium and samples were withdrawn from each well, and MTT (3-(4,5-dimethylthiazol-2-yl)-2,5-diphenyltetrazolium bromide) test was performed. This test is based on the activity of mitochondrial dehydrogenases of vital cells that convert MTT in formazan crystals. DMEM phenol red-free and 50 μL of MTT dissolution were added in each well, the final MTT concentration being 2.5 mg/mL. MTT-NHDF contact was maintained for 3 h before the whole supernatant was withdrawn and substituted by 100 µL of dimethyl sulfoxide solution (DMSO, Sigma-Aldrich^®^-Merck, Milan, Italy) to dissolve formazan purple salts. The absorbance was assayed at 570 nm by means of an ELISA plate reader (Imark Absorbance Reader, Bio-rad, Hercules, CA, USA), with a reference wavelength set at 655 nm. Cell viability was calculated as % ratio of the absorbance of each sample and the absorbance of the cells kept in contact with the growth medium (control).

#### 2.3.2. Cell Motility Assay for Wound Healing

The gap closure cell motility assay is based on the employment of a Petri μ-Dish^35 mm, low^ (Ibidi, Giardini, Italy) in which a silicone insert is enclosed. The insert comprises two chambers with a growth area of 0.22 cm^2^ divided by a septum with a width of cell-free gap of 500 ± 50 μm. NHDFs were seeded in each chamber at 10^5^ cells/cm^2^ concentration and were grown until confluence in the same conditions described in Section 2.3.1. After 24 h, fibroblasts reached confluence, and the silicone inserts were subsequently removed with sterile tweezers, displaying two areas of cell substrates divided by the 500 μm (± 50) gap.

Cell substrates were washed with sterile phosphate buffer solution (PBS; 10% *v*/*v*) to eliminate debris. Then, they were put in contact with a final volume of 700 μL of phenol red DMEM in which samples were included at determined concentrations. These concentrations were selected according to MTT results. Particularly, PS9, G30, PS9ALI, PS9GR, G30ALI and G30GR were used in concentration B (50 μg/mL of clay mineral), and spring waters were used in 2.5% *v*/*v* concentrations. Cells kept in contact with pure growth medium were used as control.

Microphotographs were taken at prefixed time intervals (0, 24, 48 h) to evaluate cell growth inside the gap. Anoptical microscope (Leica, DMI3000-B model) equipped with LAS EZ software was used (Leica microsystems, Wetzlar, Germany). In order to analyze results in a more objective way, the full area of the wound healing space was photographed in all samples. Then, wound closure was monitored by measuring the remaining gap with ImageJ software. The percentage of wound closure was calculated according to Equation (2), where WS0 stands for “wound space at time 0” and WS24 is “wound space after 24 h”, both of them measured as an area (μm^2^).

(2) $$\%\;\mathrm{Wound}\;\mathrm{closed}\;\mathrm{after}\;24\;h=100\;-\;\frac{{\mathrm{WS}}_{24}\left({\mu m}^{2}\right)\cdot 100}{{\mathrm{WS}}_{0}\left({\mu m}^{2}\right)}$$

### 2.4. Confocal Laser Scanning Microscopy

An additional sequence of wound healing experiments was stopped at 24 h of growth in order to study the morphology of fibroblasts during the wound closure procedure. NDHFs were washed three times with PBS (10% *v*/*v*) and fixed with glutaraldehyde solution in PBS (3% *v*/*v*, 800 μL; Sigma-Aldrich^®^-Merck, Milan, Italy). Contact with glutaraldehyde was maintained for 2 h (4–8 °C), and all samples were protected from light. Three PBS washes were once again performed prior to fibroblast permeabilization. Permeabilization was performed by adding Triton X-10 (0.1% *w*/*v*) for 10 min at room temperature. Triple PBS wash was again performed. Fluorescein isothiocyanate (FITC, λ_ex_ = 495 nm; λ_em_ = 513 nm)-labeled phalloidin (Phalloidin-FITC, Sigma-Aldrich) was used to mark polymerized F-actin in the cytoplasm of NHDF (50 μg/mL, darkness, 40 min at room temperature). The procedure was defined according to fabricant indications. After several PBS washings aiming to eliminate unbound phalloidin-FITC, fibroblast nuclei staining was done. Blue fluorescence nucleic acid stain 4′,6-diamidino-2-phenylindole (DAPI, Sigma-Aldrich) was used. This molecule binds to double-stranded DNA, thus labelling nuclei (λ_ex_ = 485 nm; λ_em_ = 552 nm). Contact between DAPI and cells was maintained for 10 min at room temperature in darkness. Finally, samples were washed and preserved in PBS (10% *v*/*v*) to avoid dryness. Confocal Laser Scanning Microscopy (CLSM) microphotographs were obtained by a Leica TCS SP2 (Leica Microsystems, Milan, Italy). Images were processed with ImageJ software.

### 2.5. Statistical Analysis

The statistical differences were determined by means of non-parametric Mann–Whitney (Wilcoxon) W test. In all cases, SPSS Statistic software was used, and differences were considered significant at *p*-values ≤ 0.05. Only significant differences are reported.

## 3. Results and Discussion

### 3.1. Caracterization of Inorganic Ingredients

#### 3.1.1. Particle Size

**Figure 1:**
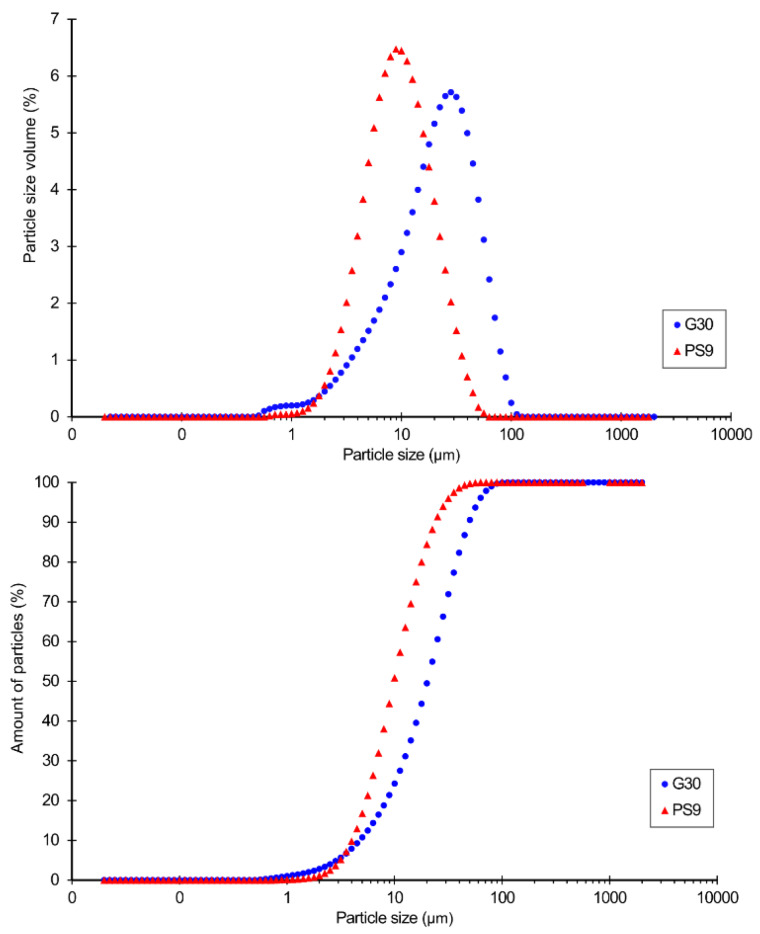
Particle size analysis of PS9 and G30. Differential analysis (up) and cumulative percentage of particles (down).

The granulometric distribution of PS9 and G30 is plotted in Figure 1. Both samples were unimodal and, therefore, homogeneous. PS9 showed to be finer than G30 (Table 4), the latter one slightly asymmetric. Calculated SPAN factors showed that the amplitude of particle size distribution for both samples had no significant difference (Table 4).

In terms of hydrogels, the finer the particles of the solid phase, the higher the stability of the resultant semisolid system (no phase separation) and the better the textural properties (smoothness). Mineralogical composition of G30 (Table 1) included 26% *w*/*w* of quartz. The presence of this mineral could influence rheology and textural properties of hydrogels. Particularly, it infers an abrasive texture to the preparation if quartz particle sizes are big. Particle sizes higher than 150 μm could be abrasive, particularly when they have remarkable hardness, such in the case of quartz [66]. Despite the presence of 26% of quartz mineral in G30, its particles were smaller than 100 μm (Figure 1), which means that resultant hydrogels possessed smooth textures, which are crucial for the acceptance of the patients [67].

#### 3.1.2. Cation Exchange Capacity

The individual exchangeable elements and total CEC of PS9 and G30 are summarized in Table 5. Total CEC values were inside the expected CEC limits for sepiolite (9.18 mEq/100 g) and palygorskite (16.29 mEq/100 g) [68,69]. Sepiolite and palygorskite CEC are usually <25 mEq/100 g [70] with higher values usually related to impurities [71,72,73,74,75,76,77,78,79,80,81]. The higher CEC showed by G30 with respect to PS9 could be explained by the presence of 6% *w*/*w* of smectites and/or sepiolite in G30, as previously described.

The main exchangeable cations for both clays were Mg^2+^ and Ca^2+^, which was in agreement with the chemical composition of PS9 and G30 showed by X-ray fluorescence analysis [63], though Na^+^ and K^+^ were also detected in small amounts. Na^+^, K^+^, Mg^2+^ and Ca^2+^ are essential elements since they are widely found inside and outside human cells [82,83,84]. The presence of suitable levels of ions such as calcium, magnesium, sodium and potassium in the wound bed are important to enhance the healing process. They allow the activity of the enzymes involved in the healing process, leading to the cascade of the repairing and regenerative processes. Specifically, calcium and magnesium levels should raise during the first 5 d of wound healing in order to promote granulation tissue formation and epidermal cell proliferation [85]. Moreover, during the restoration of the trans-epithelial potential of cells in the damaged tissue, Na^+^, K^+^ and/or Ca^2+^ play a crucial role [86]. The bioavailability of these cations in the wound site should promote the healing process and contribute to fasten the damaged area reparation.

Mg^2+^ cations, abundant in both ALI and GR, have demonstrated remarkable properties for tissue regeneration and repair, particularly in the promotion of collagen formation and angiogenesis on skin wounds [56,87]. For this reason, it is conceivable that ALI and GR should perform wound healing effects, as previously remarked. The maximum exchangeable amount of Mg^2+^ from PS9 and G30 corresponded to 17.67 and 37.14 mg/L, respectively (Table 5). Magnesium concentration due to the spring water was 5–12 mg/L, and this proved to be effective during wound healing by Sasaki et al. [56]. Consequently, both PS9 and G30 were considered as potentially effective minerals due to their exchangeable Mg^2+^ content.

Normal homeostasis of mammalian skin is also maintained by elements such as calcium, modulating keratinocyte and fibroblast proliferation and differentiation [88]. Certain skin disorders, such as psoriasis, have been related to Ca^2+^ disorders in keratinocytes [89,90]. Extracellular calcium is a determinant factor in the differentiation and maturation of fibroblasts, and its effectiveness is dose-dependent [89] Another recent study on wound healing demonstrated that calcium cations released from a calcium alginate wound dressing promoted endothelial cell growth and proliferation [91].

Zinc is also important during wound healing steps [85,88], though it was not detected as an exchangeable cation of PS9 nor G30 through the ICP-OES measurements performed. However, both ALI and GR waters contained Zn (Table 2).

Potassium has also demonstrated to favor wound healing (fibroblast differentiation, re-epithelialization, migration and proliferation of dermal cells), so its presence both in spring waters and hydrogels is considered as a positive feature [92,93].

#### 3.1.3. Zeta Potential

**Figure 2:**
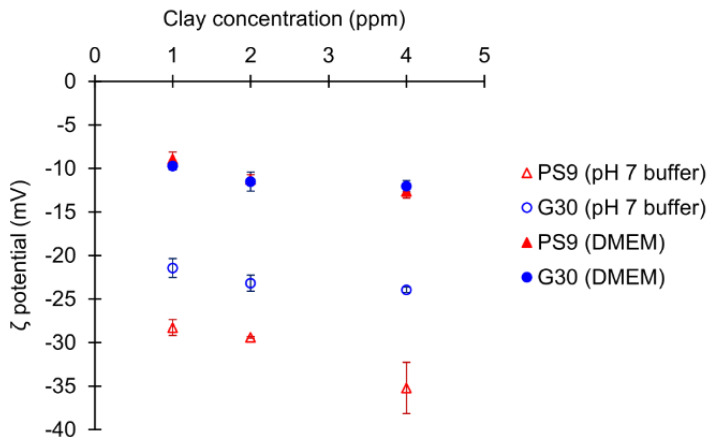
Zeta potential variations of PS9 and G30 (mean values ± s.d.; n = 3) in pH 7 buffer and complete DMEM culture medium at different concentrations.

Zeta-potential results of PS9 and G30 at different concentrations are presented in Figure 2. Regarding ζ potential of minerals in pH 7 buffer, PS9 and G30 results were in agreement with other sepiolite and palygorskite samples previously studied [94,95,96]. In particular, the zeta potential of PS9 in aqueous pH 7 solution was more negative (higher) than that of G30.

It has been demonstrated that surface charge of nanoparticles has the potential to influence cell viability [97]. The importance of clay particle zeta potentials during biocompatibility and wound healing tests lies in the fact that cells possess a negative surface zeta potential. Negative zeta potential is one of the most decisive factors of biocompatible materials, showing higher cell viability [98,99]. It is known that particles with positive zeta potentials interact and/or penetrate cells easily due to their opposite charge, thus being one of the main strategies to improve transfection efficiency [100,101]. Negatively charged particles have also proved to interact with cells up to a certain extent and even be able to enter by endocytosis-mediated mechanisms [102,103], although this happens with higher difficulty for negative particles than for positive ones. The uncontrolled entrance of certain substances into the cells could jeopardize their viability, thus inferring that positively charged materials are more likely to put the cell viability at risk.

Another factor by which nanoparticle surface charge demonstrated to influence cell viability is due to their agglomeration state [99]. Berg and co-workers revealed that hepatocytes showed less viability when exposed to metal nanoparticles with zeta potentials close to their isoelectric point [97]. This change in zeta potential was also strongly related to the agglomeration state of the very same particles. That is, the more neutral the nanoparticles are, the more easily they aggregate and, subsequently, the faster their precipitation over the cell tapestry and/or their interaction with the negatively charged cell membranes.

Cell media, such as DMEM, have demonstrated to significantly modify zeta potential of different suspended particles [104,105,106]. These changes are ascribed to the presence of a wide variety of charged molecules such as amino acids and vitamins, among others. The interaction of clay particles with these molecules changes the zeta potential of the former ones. In fact, PS9 and G30 suffered a significant change of zeta potential when added to full DMEM culture medium (Figure 2). In this occasion, no significant differences were found between PS9 and G30 values, thus confirming that the resultant surface charges of the particles is governed by the culture medium. Therefore, during biocompatibility and wound healing tests, clay particles should maintain a −10 mV zeta potential. The reduction of zeta potential strength would reduce the stability of the clay suspensions, making them less flocculated and, consequently, more prone to precipitation phenomena. Precipitation of clay particles could hinder cell viability by cell suffocation. Nonetheless, the fact that they still showed a negative surface charge would hinder the entrance of clay particles inside cells. These results will be confirmed by MTT analysis.

### 3.2. In Vitro Tests of Inorganic Ingredients, Spring Waters and Hydrogels

#### 3.2.1. Biocompatibility Tests

**Figure 3:**
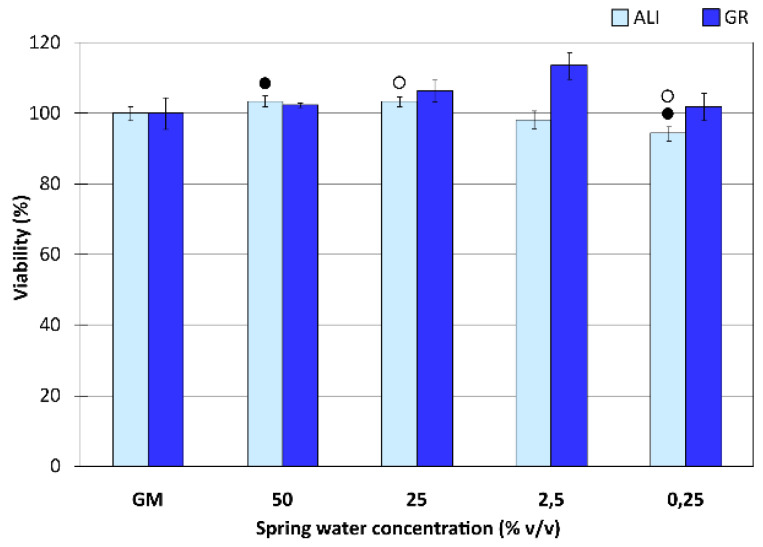
Biocompatibility tests (contact time 24 h) of ALI and GR medicinal waters. Viability (%) vs. medicinal water concentration (% *v*/*v*) toward NHDF. GM stands for “growth medium” and refers to control test. Mean values ± s.d.; n = 8. Significant differences are marked as (●) ALI 50% (*v*/*v*) vs. ALI 0.25% (*v*/*v*); (○) ALI 25% (*v*/*v*) vs. ALI 0.25% (*v*/*v*). Mann–Whitney (Wilcoxon) W tests, *p* values ≤ 0.05.

Biocompatibility results on NHDF treated with medicinal ALI and GR waters for 24 h are plotted in Figure 3. No significant differences were found, even when multiple comparisons were performed. In fact, ALI and GR viability results were around 100% for all water dilutions. Therefore, mineral medicinal waters studied in this work did not hinder dermal fibroblasts viability, thus determining their biocompatibility. Internal comparisons between ALI and GR concentrations were also performed. Mann–Whitney marked differences in ALI viability were between 50% vs. 0.25% and 25% vs. 0.25% (Figure 3). Su et al. detected an optimal dilution of a concentrated underground mineral spring water that made skin cells to grow better in comparison with other dilutions [107]. By the same token, ALI concentrations higher than 25% (*v*/*v*) could allow slightly better NHDF growth performances with respect to more diluted samples.

**Figure 4:**
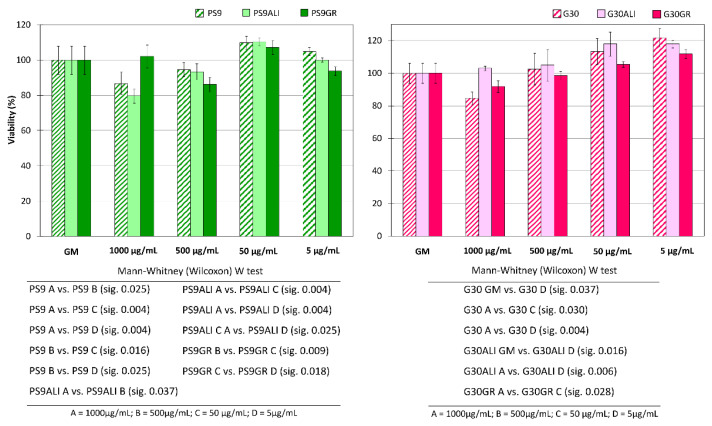
Biocompatibility tests (contact time 24 h) of PS9, PS9ALI and PS9GR (left) and G30, G30ALI and G30GR (right). Viability (%) vs. clay concentrations toward NHDF. GM stands for “growth medium” and refers to control test. Mean values ± s.d.; n = 8. Significant differences were reported within the figure (down) as “sig” which refers to “*p*-values”.

PS9 clay mineral and corresponding peloids PS9ALI and PS9GR are plotted in Figure 4 (left) as well as G30 and its corresponding peloids G30ALI and G30GR Figure 4 (right). In a general view, the lower the sample concentration (either clay or thermal mud), the higher the cell viability. None of the samples nor their concentrations presented a cellular viability below 80%. This fact indicated the absence of drastic cellular cytotoxicity within 24 h. With respect to PS9 and G30 clays, it was possible to observe a slight reduction of the cellular viability (with respect to GM) as the clay concentration increased. In fact, when the clay mineral concentration corresponded to 1 mg/mL, cellular viability was close to 80% (for both pristine clays and thermal muds). Nonetheless, statistical analyses indicated no significant differences between PS9 and G30 1000 μg/mL concentrations vs. GM, which confirmed that PS9 and G30 were highly biocompatible against NHDF cells. G30 5 μg/mL concentration showed significant differences vs. GM. Notably, the lower the concentration of the clay, the better the cellular proliferation. It has been suggested by some studies that the precipitation of clay minerals could hinder cell viability by some sort of physical effect [50,103]. The biocompatibility of Veegum HS © (Bentonite clay) was evaluated by Salcedo et al., finding that at 167 μg/mL of Veegum HS © concentration the viability of Caco-2 cells reduced approximately to 50% [50]. This effect was ascribed to the precipitation of clay particles, which blocked cell membrane channels. According to the authors, montmorillonite had a particle size ranging from 45–75 μm [50], which is a particle size range very similar to that of PS9 and G30. In these conditions, it was argued that if the precipitation of clay particles occurred, cell viability could be compromised at concentrations ≥160 μg/mL. On the contrary, neither PS9 nor G30 showed fibroblasts viabilities lower than 50% even at the highest concentration tested, thus demonstrating to possess a remarkable biocompatibility.

Internal statistical studies for PS9 and G30 results detected significant differences in G30 1000 μg/mL vs. 50 μg/mL and 5 μg/mL. For PS9, concentrations of PS9 1000 and 500 μg/mL differed both between them as well as with 50 and 5 μg/mL (see Figure 4). These findings mean that, for both clays, smaller clay amounts in contact with fibroblasts were characterized by a higher degree of fibroblast biocompatibility. The biocompatibility results obtained for both clays were in agreement with the existing literature. Sepiolite clay mineral (Pangel S9) has been previously tested by Fukushima et al. towards fibroblasts and osteoblasts [108]. In this case, authors combined 10% of sepiolite with poly(butylene adipate-co-terephthalate) reporting no cytotoxicity. A Tolsa’s Group sepiolite clay was also employed by Fernandes et al. [109]. In this case, they reported a reduction of HeLa cell viability of 50% when sepiolite was present in 1000 µg/mL concentration. No such a reduction was found in this work when PS9 was used at the same concentration. Nonetheless, these differences could be ascribed to differences in the type of cell culture [110]. Apart from anti-inflammatory, antibacterial and anti-oxidant properties of a natural Spanish palygorskite, its cytotoxicity against murine macrophages has been reported to start from 300 µg/mL onwards [58,59]. Particularly, authors reported a reduction of 20% viability at the aforementioned concentration. Once again, in the present study, G30 showed higher biocompatibility (at least against fibroblasts) because, even at 500 µg/mL concentration, fibroblasts did not show a viability reduction. In more recent studies, pharmaceutical grade palygorskite (Pharmasorb^®^ colloidal) was able to protect human dermal fibroblasts against carvacrol cytotoxicity at clay concentrations ranging from 8 to 12 µg/mL [6].

Neither PS9ALI nor PS9GR showed significant differences between GM and tested concentrations, thus demonstrating the total biocompatibility of these hydrogels. Statistically, PS9ALI and PS9GR thermal mud showed similar significant differences between concentrations, following the same trend previously described for PS9. In fact, 50 and 5 μg/mL clay concentrations apparently favored cellular viability (Figure 4, left). The agreement among PS9ALI, PS9GR and PS9 biocompatibility results allowed the confirmation of their reproducibility. Both PS9ALI and PS9GR, as well as PS9, shared significant differences of concentration 50 μg/mL due to the higher viability results obtained at this concentration (Figure 4, left).

Regarding G30ALI and G30GR peloids, Mann–Whitney statistical analysis pointed out significant discrepancy in activity between G30ALI GM and 1000 μg/mL concentration vs. 5 μg/mL and between G30GR 1000 μg/mL vs. 50 μg/mL (as specified in Figure 4, right). Since the same trend has been reported for G30 samples, once again it was possible to confirm the reproducibility of the results.

Despite the complexity generated by the statistical analysis, all solid samples (Figure 4) showed a better result for 50 µg/mL concentration, always with cellular viabilities higher than 100%. According to these findings, 50 µg/mL clay concentration was the safest/most ideal one for fibroblast cultures, regardless of the type (PS9 or G30) or the origin of the clay (powder or thermal mud). It is also important not to forget the fact that all the tested concentrations were biocompatible for fibroblasts within 24 h of contact. Due to the conclusions reached with the MTT test, 50 µg/mL was the clay concentration selected for further studies including cell motility assay (wound healing) and proliferation CLSM tests. For spring waters, 50% *v*/*v* concentration was used.

#### 3.2.2. Cell Motility Assay for Wound Healing

**Figure 5:**
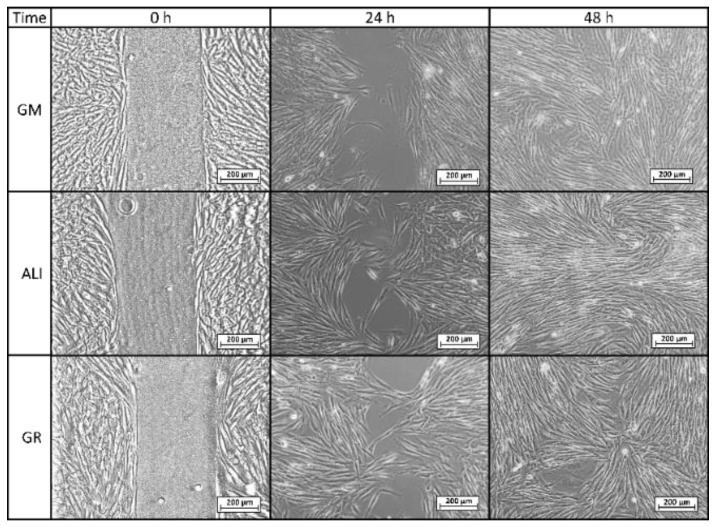
Optical microscopy of wound healing tests for control group (GM) and spring waters (ALI and GR) at 50% (*v*/*v*) culture concentration.

**Figure 6:**
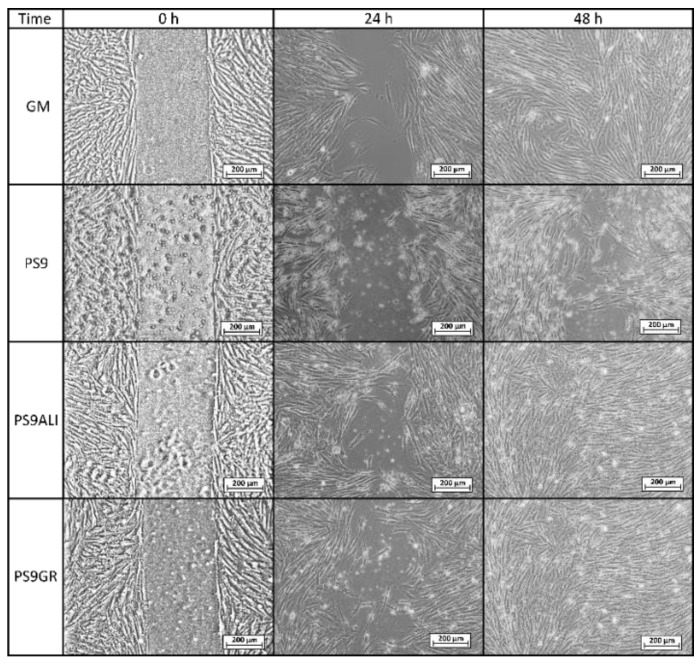
Optical microscopy of wound healing tests for control group (GM), PS9 clay mineral and corresponding peloids PS9ALI and PS9GR, all of them with a clay concentration of 50 μg/mL.

**Figure 7:**
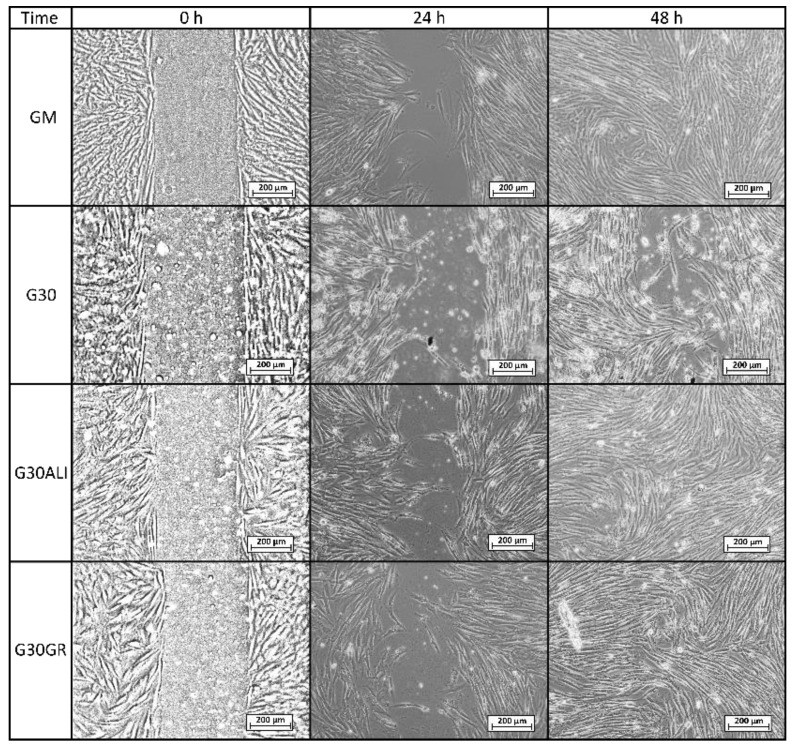
Optical microscopy of wound healing tests for control group (GM), G30 clay mineral and corresponding peloids G30ALI and G30GR, all of them with a clay concentration of 50 μg/mL.

**Figure 8:**
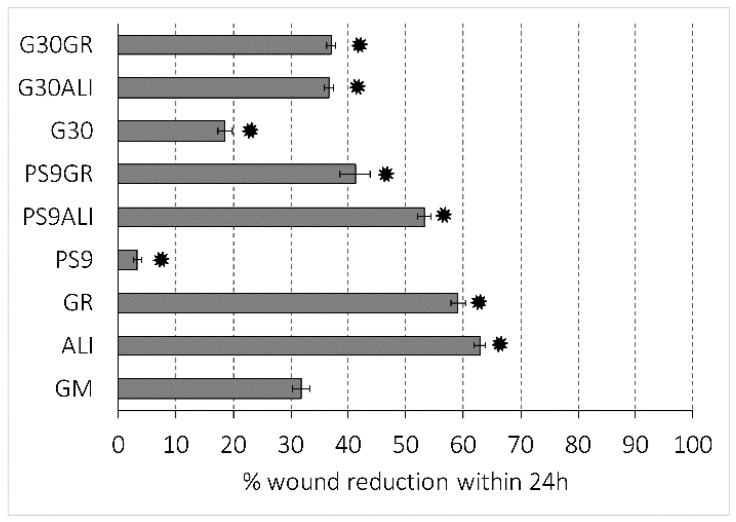
Histogram of % wound reduction after 24 h, calculated according to Equation (2). Mean values ± s.d. (n = 6). Significant differences between samples and GM are marked with *.

Microscopy images of gap closure results are reported in Figure 5, Figure 6 and Figure 7. ALI and GR samples were tested at 50% (*v*/*v*) concentration, while PS9, G30, PS9ALI, PS9GR, G30ALI and G30GR were tested at 50 μg/mL of clay mineral. The microphotographs taken at zero time (0 h) showed for all the samples the presence of defined gaps mimicking wounds. Normal fibroblasts grown at confluence were clearly visible in each side of the wound. The time 0 gaps were reported to measure approximately 500 µm, which was in agreement with the variability of the silicone inserts of the Petri μ-Dishes used (500 ± 50 µm). Insoluble clay particles were visible in the cultures treated with PS9, G30 and hydrogels PS9ALI, PS9GR, G30ALI and G30GR, unlike GM, ALI and GR. In all cases, after 24 h, fibroblasts started to invade the gap and even established contact with the cells of the opposite side in certain points of some samples. NHDF cells also maintained their typical fusiform morphology during the whole experiment. These facts confirmed that the presence of spring waters, clay or hydrogels did not impair cell growth. This was in line with the biocompatibility results previously discussed. In all cases, fibroblasts crossed the empty zone after 48 h, thus forming anastomosis. In spring water ALI and GR samples, fibroblasts of both sides of the wound established contact within 24 h (Figure 5). In comparison with the control (GM), it was clear that the addition of ALI and GR favored wound closure, since the contact points between fibroblasts were more numerous. According to the measurements of the wounded area performed by image analysis, ALI and GR samples covered, respectively, 62.9% and 59.1% of the initial wounded area, whereas GM covered about 31.8% of the wound (Figure 8). After 48 h all the cell substrates reached confluence, and this further supports the biocompatibility of the hydrogels and their components in vitro. Previous works have claimed that the presence of elements such as B^3+^ and Mn^+2^ in keratinocyte culture mediums induced the migration of these cells [30]. They tested boron and manganese at different concentrations (500–1000 μg/L and 100–1500 μg/L, respectively). In fact, keratinocytes in contact with these elements were able to cover the artificial gap (scratch-assay method) with 20% more efficiency with respect to the control group (without B^3+^ and Mn^+2^) in 24 h. ALI and GR chemical compositions have been previously reported by Aguzzi et al. [111] and García-Villén et al. [63] (Table 3). The absence of significant differences between GR, ALI and GM groups further confirmed the biocompatibility of both mineral medicinal waters since they did not hinder wound closure. The presence of B^3+^ and Mn^+2^ in both GR and ALI could explain why they did not interfere with cell motility during wound healing in a significant manner. Other elements such as As^3+^ and Fe^2+^ (both presents in ALI and GR waters) have also demonstrated to improve wound healing, particularly of the nasal mucosa [112]. General skin regenerative properties of spring waters have been reported in the literature [32,33,34,113]. The study of Liang et al. demonstrated that skin regenerative properties of Nagano spring water were related to the spring water chemical composition, with no influence of microorganisms [31]. Another beneficial effect that could be ascribed to ALI and GR according to their chemical composition is the protective effect against oxidative stress due to the presence of sulfur [114].

Regarding PS9 and G30 clay samples (Figure 6 and Figure 7), it was not possible to find zones with full fibroblast confluence after 48 h, though both sides of the wound were able to establish contact at this time. Fibroblast contacts of PS9 and G30 samples in the culture medium were slower with respect to GM. According to MTT results (Section 3.2.1), which were also performed for 24 h, pristine clays did not hinder cell viability. However, their presence seemed to impede fibroblast mobility during the wound healing assay, slowing down the total gap closure with respect to GM and studied peloids. In fact, the uncovered gap width after 24 h was significantly higher for PS9 and G30 with respect to the rest of the samples (Figure 8), thus indicating a slower coverage of the wounded area. For these similar samples, the majority of insoluble particles visible within the cell substrate were concentrated adjacent to or over/inside fibroblasts. It is well known that cell membranes are negatively charged and can be penetrated by positive substances. Negatively charged particles can also interact and even penetrate cells by endocytosis-mediated mechanisms [102,103,115]. According to the zeta potential results previously reported (Figure 2), PS9 and G30 had a negative net charge in the major part of the pH range tested. These clay particles could interact between them (thus forming bigger aggregates) and with cells by establishing interactions such as Van der Waals forces. This hypothesis was the starting point of Abduljauwad and Ahmed, who proposed that the interaction between clay minerals and cells would hinder cell migration [116]. They evaluated the roles of montmorillonite, hectorite and palygorskite particles in cancer cell migration and demonstrated these materials could prevent cellular metastasis. Results of scratch-induced wound healing assays reported by these authors demonstrated that clay mineral particles significantly delayed the gap closure in comparison with control experiments. For instance, while the control showed full gap closure within 24 h, palygorskite showed a mean gap closure of 59 ± 3% after 24 h. Polymeric composite films containing montmorillonite were evaluated by Salcedo et al. [50] and Mishra et al. [117]. The scratch assay showed, in both cases, that the presence of montmorillonite particles alone was able to alter cell behavior (Caco-2 and fibroblasts, respectively for each study), slowing down the gap closure. Similar results were reported by Vaiana et al. on montmorillonite and keratinocytes [118]. These results were in agreement with PS9 and G30 performances during wound healing: though clays were not cytotoxic, more time would be necessary to obtain complete cell confluence within the artificial wound (gap) in their presence. The biocompatibility and safety of natural clay minerals such as palygorskite during wound healing was also supported by in vivo studies. A natural Brazilian palygorskite was tested for in vivo wound healing of rats [119]. In comparison with functionalized clay minerals, the natural one provided more advanced and safer wound healing, since histological cuts demonstrated the presence of dermal papilla and hair follicles after 14 d of treatment [119].

The presence of hydrogels promoted in vitro fibroblast mobility during wound healing processes. In fact, coverage of the artificial gap was faster for PS9ALI, PS9GR, G30ALI and G30GR with respect to GM and pristine clays (Figure 8). It is worth to note that all therapeutic muds were used in concentrations equal to the powdered clay samples. That is, the amount of peloid within the culture medium was higher in order to compensate for them only possessing a 10% *w*/*w* of clay mineral. Whatsoever the nature of the interaction between clay particles and fibroblasts, responsible for the gap closure deceleration (observed in PS9 and G30 tests), it was reduced to a minimum when it came to thermal mud formulations. In fact, significant differences were found between gap closures of peloids vs. GM, demonstrating that the evaluated hydrogels induced a positive effect during wound healing.

These results are supported by previous studies in which clay minerals have reported to exert neutral or positive wound healing effects when combined with other substances [44,120]. For instance, no significant changes have been found during in vitro wound healing studies of montmorillonite–chitosan–silver sulfadiazine nanocomposites with respect to the control. That is, the presence of the clay mineral did not hinder the gap closure procedure and did not affect fibroblast phenotypes [54]. On the other hand, better skin re-epithelialization and reorganization have been found when halloysite and chitosan were combined to form a nanocomposite with respect to the use of both materials independently [47]. In vivo infected wound treatment with silver nanoparticles was improved by the use of montmorillonite as a carrier, which reduced the drug cytotoxicity [121]. Montmorillonite also demonstrated to exert a wound healing effect when combined with chitosan and polyvinylpyrrolidone polymers [48]. The nanocomposite films containing bentonite increased the in vivo wound healing processes in mice when compared with the formulations without the clay. For instance, wound closure after 16 d was 92–93% for samples without clay and 95–97% for those with montomorillonite, and all of them were higher than the negative control at the same time (84%) [48].

Despite the scarce literature regarding the use of therapeutic muds in skin regenerative properties, the existing studies were also in agreement with the present observations. Thermal mud treatments with volcanic deposits of Azerbaijan facilitated the healing of chronic gangrenous wounds of diabetic patients [60]. The major part of the patients subjected to pelotherapy reached total wound recovery by the end of the treatment. Two natural Dead Sea black mud samples were evaluated by Abu-al-Basal for their in vivo wound healing properties [61]. One of them was used in its pristine state, while the other was formulated in form of a facial mask by adding plant extracts and vitamin E. Both samples accelerated the wound healing process by enhancing granulation, wound contraction, epithelialization, angiogenesis and collagen deposition. Another in vivo wound healing study was performed by Dário et al. [62]. They evaluated a natural peloid extracted from Ocara Lake (northeast of Brazil), which was sieved, solid-state characterized and sterilized. The solid fraction was mainly composed of quartz, illite and kaolinite, and it was formulated as an emulsion. The emulsion was put in contact with injured Wistar rats. The formulation including the Ocara Lake solid fraction induced histological changes in the deep dermis that allowed an effective healing with respect to control groups. On the other hand, there is evidence in literature about the anti-inflammatory properties of peloids [122]. Moreover, a recent study focusing on fibrous clays suggests that sepiolite and palygorskite, the main components of PS9 and G30, respectively, did not influence the in vitro NO (inflammation signal) production of murine macrophages (RAW 264.7). Furthermore, both clays decreased the leucocyte infiltration shortly after exposure in vivo on mouse ear edema (12-O-tetradecanoylphorbol-13-acetate (TPA) as inflammatory agent). In particular, sepiolite and palygorskite caused decreases on the number of infiltrated cells per field [59]. Analogously, other clay minerals, as halloysite and montmorillonite, proved macrophage cytocompatibility and negligible secretion of TNFa, as proinflammatory cytokines [123]. The anti-inflammatory properties of clays could control the inflammatory phase in the healing process, shortening healing time and avoiding wound chronicity.

### 3.3. Confocal Laser Scanning Microscopy

**Figure 9:**
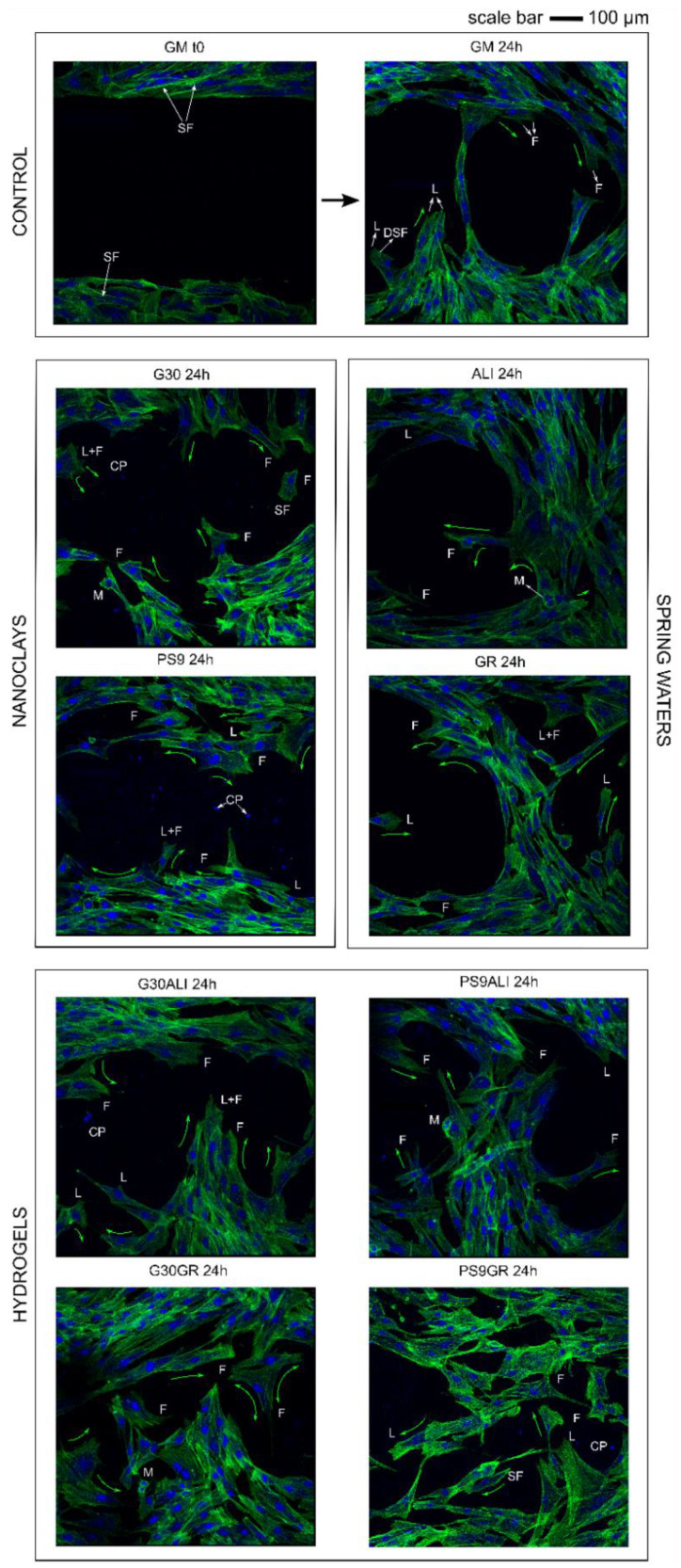
CLSM microphotographs during wound healing. NHDMs were stained with phalloidin-FITC (green, F-actin filaments) and DAPI (blue, nucleus). Green arrows indicate the migration direction; different F-actin structures have been identified by L (lamellipodia), F (filopodia), SF (stress fibers) and DSF (dorsal stress fibers). Additionally, M stands for (mitosis) and CP stands for (clay particles).

CLSM microphotograph results (Figure 9) showed fluorescence of fibroblast nuclei and cytoplasm. Green fluorescence is due to the binding of phalloidin specifically with F-actin, and the blue one is due to 4’,6-diamidino-2-phenylindole (DAPI) bounded to DNA. Morphology of fibroblasts during wound healing is very important, since it gives information about migration and adhesion of cells. F-actin filaments facilitate cell–cell interaction and tissue regeneration [124]. In all samples, regardless of the type of sample with which fibroblasts were put in contact with, NHDF showed typical fusiform morphology. Moreover, fibroblasts located in the wounded area possessed typical morphology of migrating cells. Therefore, it was possible to identify retraction/protrusion parts of fibroblasts due to the position of F-actin. Typical actin filament structures were detectable in all samples: lamellipodia, filopodia and stress fibers (dorsal stress fibers and transverse arcs). Moreover, fibroblasts in mitosis were also visible in some points.

Regarding the speed of wound closure, the CLSM results were in agreement with those obtained by optical microscopy. GM control after 24 h demonstrated that NHDFs were able to make contact and started to close the gap, though yet some empty zones were clearly visible. The number of cells inside the wound gap in ALI and GR samples was higher than that in GM. PS9 and G30 showed the worst result in terms of “gap closure”, which confirmed the previous results. Once again, it was possible to observe that the “slowing down” effect of PS9 and G30 did not happen when both clays were formulated in form of nanoclay/spring water hydrogels. Particularly, hydrogels G30ALI, G30GR, PS9ALI and PS9GR induced a faster wound healing with respect to the rest of the samples.

## 4. Conclusions

Fibroblast biocompatibility and wound healing efficacy of inorganic hydrogels formulated with two nanoclays in two natural spring waters have been studied. Both spring waters were fully biocompatible and favored wound healing, inducing faster gap closure with respect to control. The studied nanoclays did not interfere with cell viability to a great extent (≥80% of cell viability), thus not being cytotoxic at the studied concentrations. Nonetheless, they interfered with the in vitro wound healing processes, slightly delaying gap closure when used as powders. This effect could be ascribed to the presence of non-flocculated nanoclay particles in the culture medium. Hydrogels formulated with the aforementioned ingredients did not hindered gap closure and reported a higher percentage of wound closure after 24 h with respect to the control.

In conclusion, this study has demonstrated the usefulness and potential of PS9 and G30 clay minerals as excipients in the preparation of hydrogels intended for wound healing and other therapeutic uses. Maximum in vitro wound healing effects were achieved by using PS9 in the formulation of the hydrogels. These promising results encourage the use of clay minerals as wound healing ingredients. Thermal center treatments are mostly focused on the physical effects of thermal muds, which are very effective against musculoskeletal disorders. Nonetheless, the findings of this study have opened new perspectives, since the addressed nanoclay/spring water hydrogels could also be used to treat chronic wounds.

## Figures and Tables

**Table 1:** Fibrous clay (PS9 and G30) mineralogical composition (modified from [63]).

	PS9		G30	
Mineralogical Composition	Sepiolite	92%	Palygorskite	58%
	Muscovite	8%	Quartz	26%
			Fluorapatite	7%
			Smectites and sepiolite	6%
			Calcite/dolomite	3%

**Table 2:** Medicinal water characteristics and elemental composition determined by means of ICP-OES and ICP-MS (modified from [63]).

		ALI	GR
	pH ± s.d.	7.90 ± 0.0472	8.02 ± 0.0823
	Conductivity (µS/cm) ± s.d.	2251.5 ± 6.74537	2465.5 ± 8.89482
**Elemental Composition**	Ca (mg/L)	348.00	460.00
	Mg (mg/L)	109.0	88.00
	Na (mg/L)	57.00	27.40
	K (mg/L)	4.60	6.80
	B (µg/L)	25.00	12.00
	Ba (µg/L)	18.80	13.00
	Cr (µg/L)	4.3	1
	Zn (µg/L)	464.99	301.08
	Mn (µg/L)	<1	108.66
	Li (µg/L)	244.2	65.00
	Ni (µg/L)	9.4	5.20
	Fe (µg/L)	6.00	21.00
	Se (µg/L)	2.3	1.00

**Table 3:** Thermal muds tested, identification codes and composition.

Identification Code	Composition
PS9ALI	10% *w*/*w* PS9, 90% *w*/*w* ALI
PS9GR	10% *w*/*w* PS9, 90% *w*/*w* GR
G30ALI	10% *w*/*w* G30, 90% *w*/*w* ALI
G30GR	10% *w*/*w* G30, 90% *w*/*w* GR

**Table 4:** Statistical particle diameters, SPAN factor (average ± s.d.; n = 3) and main modes of PS9 and G30 clay minerals.

	PS9	G30
d_10_ (µm)	4.0 ± 0.07	4.8 ± 0.03
d_50_ (µm)	9.9 ± 0.15	20.2 ± 0.03
d_90_ (µm)	23.9 ± 0.2	49.3 ± 0.10
SPAN Factor	2.0 ± 0.02	2.2 ± 0.01
Main Mode (µm)	8.9	28.3

**Table 5:** CEC results of PS9 and G30 (average mEq/100 g ± s.d.; n = 3).

mEq/100g	PS9	G30
Na^+^	0.70 ± 0.045	1.68 ± 0.071
K^+^	0.33 ± 0.023	0.13 ± 0.032
Mg^2+^	3.62 ± 0.341	7.61± 0.326
Ca^2+^	4.53 ± 0.123	6.87± 0.186
Total	9.18	16.29
